# Antibody-based therapeutics and therapeutic development for diabetic retinopathy: targeting VEGF, Ang/Tie, and inflammatory pathways

**DOI:** 10.3389/fendo.2025.1710376

**Published:** 2026-01-12

**Authors:** Yuan Zong, Shuang Qiu, Huang Zhang, Jiaxin Wu, Jingheng Du, Koju Kamoi, Zhimin Cen

**Affiliations:** 1 Department of Ophthalmology, Zhongshan Torch Development Zone People`s Hospital, Zhongshan, Guangdong, China; 2 Department of Ophthalmology & Visual Science, Graduate School of Medical and Dental Sciences, Institute of Science Tokyo, Tokyo, Japan; 3 International Ocular Surface Research Center, Institute of Ophthalmology, and Key Laboratory for Regenerative Medicine, Jinan University Medical School, Guangzhou, China; 4 Department of Ophthalmology, The First Affiliated Hospital of Jinan University, Guangzhou, China

**Keywords:** angiopoietin, anti-VEGF therapy, diabetic retinopathy, dual VEGF/Ang-2 blockade, macular edema, monoclonal antibodies, Tie2

## Abstract

Diabetic retinopathy (DR), a leading cause of global blindness, represents a significant microvascular complication of diabetes mellitus. This comprehensive review examines the evolving landscape of monoclonal antibody (mAb) therapy in DR management. The pathogenesis of DR involves complex molecular mechanisms including VEGF overexpression, angiopoietin dysregulation, inflammatory processes, and oxidative stress. The angiopoietin–Tie (Ang/Tie) axis is a master regulator of endothelial stability; Ang-2–driven suppression of Tie2 promotes vascular leak, pericyte dropout, and leukocyte adhesion, providing a mechanistic rationale for Ang-2 inhibition and dual VEGF/Ang-2 blockade. Anti-VEGF mAbs (bevacizumab, aflibercept, ranibizumab) have revolutionized DR treatment by effectively targeting neovascularization and vascular permeability. Recent clinical innovations include ophthalmic formulations of bevacizumab, high-dose aflibercept, the ranibizumab port delivery system, and bispecific antibodies like faricimab that simultaneously target VEGF and angiopoietin-2 pathways, alongside emerging preclinical investigations into novel targets and bio-engineered delivery platforms. While anti-inflammatory mAbs targeting IL-6, IL-17A, and IL-1β show theoretical promise, clinical evidence supporting their efficacy remains limited, positioning them as agents under therapeutic development rather than established care. Despite therapeutic advances, significant challenges persist, including cost-effectiveness concerns, treatment burden, and adherence issues. This review highlights the transformative impact of mAb therapy in DR management while acknowledging the need for continued innovation to address existing limitations and optimize patient outcomes through personalized treatment approaches.

## 1 Introduction

Diabetes mellitus (DM) represents a spectrum of metabolic dysregulations characterized by sustained hyperglycemia, precipitating multi-organ pathologies. Among these sequelae, diabetic retinopathy (DR) persists as the preeminent microvascular complication and a primary driver of visual impairment among the working-age population globally (1, 2). The escalating global burden of diabetes parallels the rising incidence of DR. A 2024 meta-analysis estimating the global epidemiology delineates a stark reality: approximately 1.07 million individuals suffered blindness attributable to DR in 2020, with an additional 3.28 million enduring moderate-to-severe vision impairment (MSVI) (3). Geographic stratification reveals disproportionate impacts, with the highest blindness rates observed in Latin America and the Caribbean, while North Africa and the Middle East exhibit the highest prevalence of MSVI. Such data underscores the urgent necessity for optimized therapeutic interventions.

**Figure 1:**
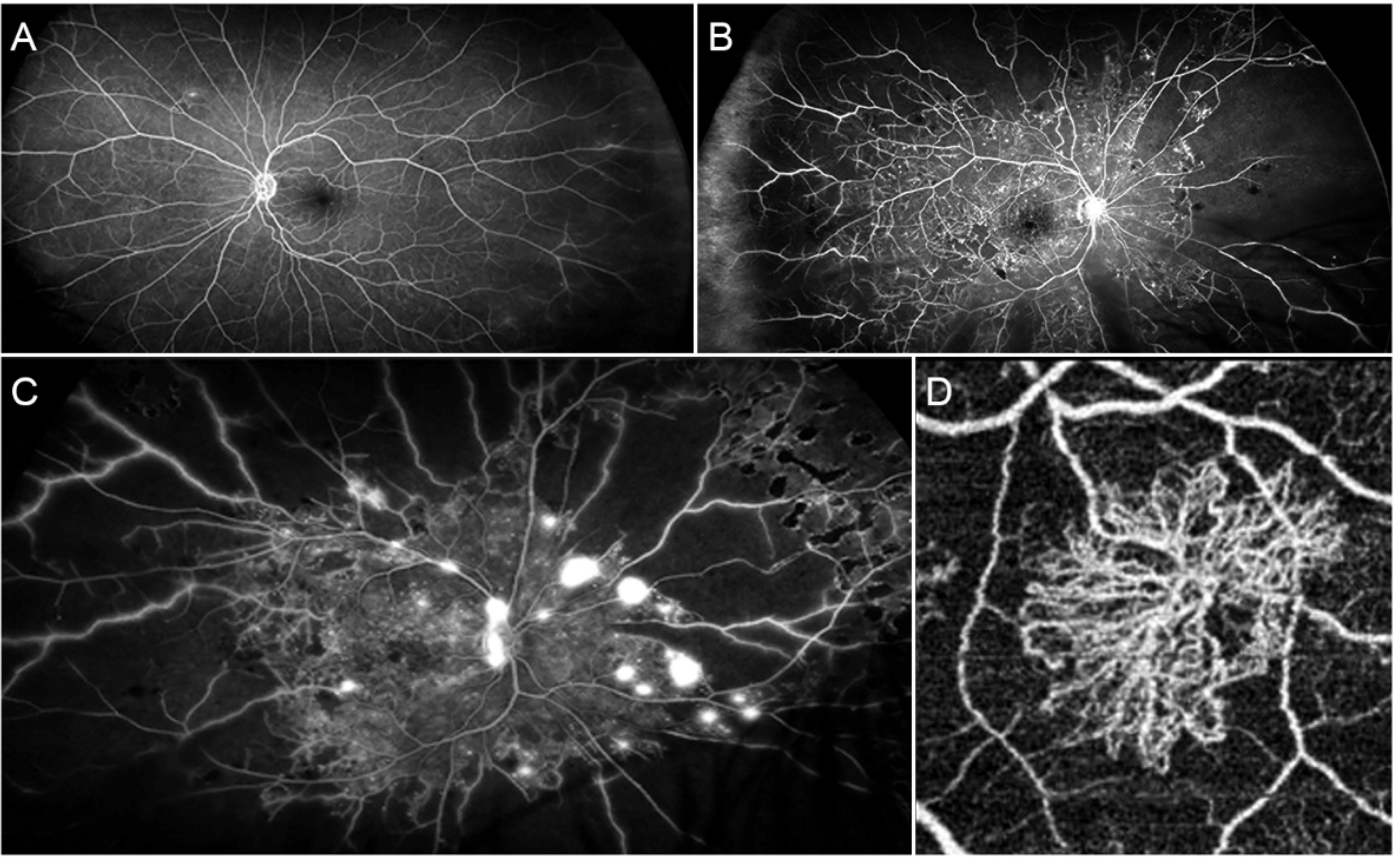
Angiographic manifestations of diabetic retinopathy at various stages: **(A)** In mild non-proliferative diabetic retinopathy (NPDR), the posterior pole architecture remains well-preserved, with only subtle capillary non-perfusion observed in the peripheral regions. **(B)** Advanced NPDR demonstrates extensive peripheral capillary non-perfusion with central involvement, exhibiting multiple microaneurysms and significant macular edema on imaging. **(C)** In proliferative diabetic retinopathy (PDR) cases, the peripheral areas reveal widespread capillary non-perfusion, while neovascularization can be detected both at the optic disc and along the retinal periphery. **(D)** Vascular reconstruction through optical coherence tomography (OCT) imaging clearly delineates the neovascular structures associated with PDR. (adapted with permission from Arrigo et al., 2022, Ref. (4), CC BY 4.0 License; see https://creativecommons.org/licenses/by/4.0/; accessed on 25 June 2025).

Clinical stratification, governed by the Early Treatment Diabetic Retinopathy Study (ETDRS) criteria, delineates DR into non-proliferative (NPDR) and proliferative (PDR) stages. (**Figure 1**) NPDR manifests through structural microvascular aberrations including microaneurysms and intraretinal hemorrhages, whereas PDR is defined by pathological neovascularization driven by retinal ischemia—a process culminating in vitreous hemorrhage and tractional retinal detachment (5). Concurrently, diabetic macular edema (DME), precipitated by the breakdown of the blood-retinal barrier (BRB), constitutes the predominant cause of central vision loss irrespective of DR severity (6).

While systemic management of hyperglycemia and hypertension remains foundational, established ocular interventions—ranging from laser photocoagulation to vitrectomy—possess inherent limitations regarding invasiveness and collateral tissue damage. The advent of monoclonal antibody (mAb) therapeutics, specifically targeting vascular endothelial growth factor (VEGF), has revolutionized management paradigms (7). Yet, the prevalence of non-responders and the burden of therapeutic regimen adherence necessitate a granular elucidation of DR pathophysiology to identify novel therapeutic targets. Accordingly, this review systematically synthesizes the clinical landscape of monoclonal antibody therapeutics in DR management, prioritizing the evaluation of established anti-VEGF agents, the strategic targeting of the Ang/Tie axis, and the investigational potential of anti-inflammatory biologics. Complementing this analysis, the manuscript delineates emerging targeted therapies and explores future perspectives regarding the optimization of mAb-based intervention strategies.

## 2 Methods

This review evaluates the therapeutic applications of monoclonal antibodies (mAbs) in diabetic retinopathy (DR) management. We conducted a comprehensive literature search using PubMed, Web of Science, and Cochrane Library databases, with the search period extending through June 2025. Key search terms included “monoclonal antibody,” “diabetic retinopathy,” “anti-VEGF,” “anti-inflammatory,” “angiopoietin,” “faricimab,” “ranibizumab,” “bevacizumab,” “aflibercept,” and “teprotumumab.” Additional relevant publications were identified through reference lists of retrieved articles, U.S. Food and Drug Administration (FDA) approval documents for pertinent therapeutics, and clinical trial registries (e.g., ClinicalTrials.gov).

Inclusion criteria encompassed English-language publications focusing on mAb-based interventions for DR, including clinical trials, observational studies, and review articles. Studies not directly addressing mAbs or DR were excluded. Data extraction emphasized study characteristics, treatment regimens, clinical outcomes (visual acuity improvement, central retinal thickness changes, DR severity score alterations), and safety profiles.

The evidence was synthesized narratively, with mAbs categorized by mechanism of action into anti-VEGF agents, anti-inflammatory agents, anti-angiopoietin agents, and other targeted therapies. Future research directions are also discussed.

## 3 The pathological mechanisms of DR

**Figure 2:**
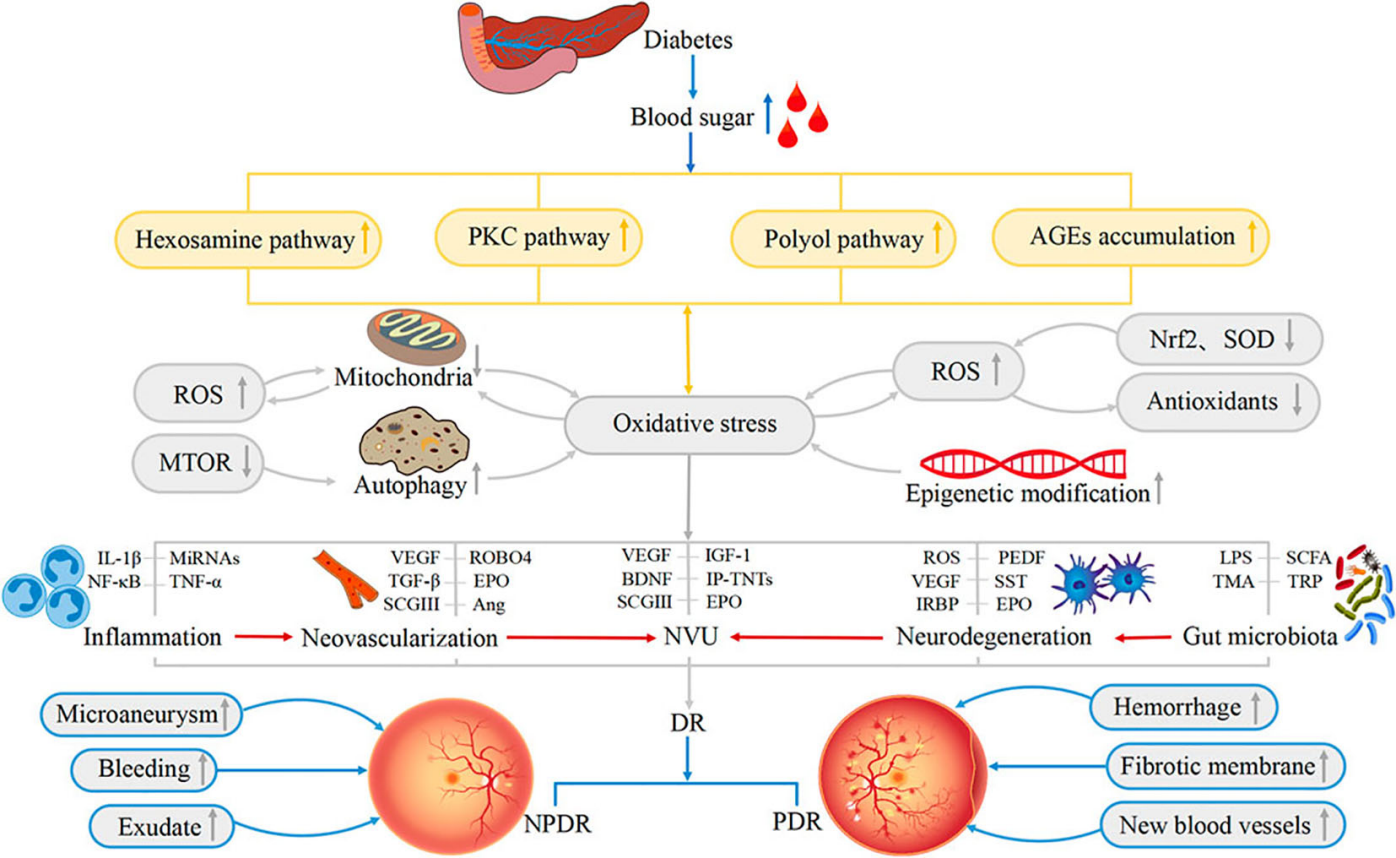
Schematic representation of the pathophysiological mechanisms underlying diabetic retinopathy (DR). Hyperglycemia activates the hexosamine, PKC, and polyol pathways, alongside AGE accumulation, precipitating oxidative stress. This oxidative burden interacts with mitochondrial dysfunction and autophagy to drive inflammation, neovascularization, neurodegeneration, NVU impairment, and gut microbiota dysbiosis. Key mediators (e.g., ROS, VEGF, SCGIII, IL-1β, LPS) orchestrate the progression from early retinal lesions (microaneurysms, exudates) to severe complications (neovascularization, hemorrhage), highlighting the multifactorial nature of DR pathogenesis.(adapted with permission from 10, Ref. (10), CC BY License; see https://creativecommons.org/licenses/by/4.0/; accessed on 25 June 2025).

Contemporary understanding posits DR not merely as a microangiopathy but as a complex neurovascular disorder, where neuroglial impairment and inflammation orchestrate disease progression alongside vascular dysfunction (8, 9), (**Figure 2**).

### 3.1 Neurovascular unit impairment and incipient neurodegeneration

The Neurovascular Unit (NVU)—an intricate functional coupling of neurons, glia (Müller cells, astrocytes, microglia), and vascular cells—maintains retinal homeostasis and BRB integrity (10). Chronic hyperglycemia instigates metabolic dysregulation via the hexosamine, polyol, and protein kinase C (PKC) pathways, alongside the accumulation of advanced glycation end-products (AGEs). These metabolic aberrations trigger oxidative stress (characterized by ROS accumulation and suppression of antioxidant defenses like Nrf2 and SOD), which in turn activates pro-inflammatory pathways (e.g., NF-κB), establishing a vicious cycle that precipitates NVU dysfunction (10, 11). Evidence substantiates retinal neurodegeneration—manifesting as retinal ganglion cell apoptosis and nerve fiber layer thinning—as an incipient event in DR pathogenesis, frequently antedating clinically detectable microvascular lesions (9, 12). Reactive gliosis within Müller cells and microglial activation result in the secretion of neurotoxic and pro-inflammatory mediators (e.g., Interleukin (IL)-1β, alongside the downregulation of neuroprotective factors such as pigment epithelium-derived factor (PEDF) and BDNF, Tumor necrosis factor (TNF)-α), which compromise the BRB and facilitate vascular permeability prior to neovascular onset (10, 13, 14).

### 3.2 The ischemic cascade and VEGF upregulation

VEGF functions as the central mediator of pathological angiogenesis in PDR and DME. Clarifying the causality of VEGF upregulation is critical: VEGF overexpression constitutes a compensatory, albeit maladaptive, response to retinal ischemia and hypoxia driven by capillary non-perfusion, rather than a direct consequence of acellular capillaries per se (15, 16). Progressive pericyte dropout and endothelial apoptosis culminate in capillary occlusion and the formation of acellular capillaries, inducing widespread retinal ischemia. This hypoxic microenvironment stabilizes hypoxia-inducible factor-1α (HIF-1α), which translocates to the nucleus to upregulate angiogenic gene expression, predominantly VEGF-A and erythropoietin (EPO) (10, 17). While physiologically intended to restore oxygenation, excessive VEGF signaling disrupts tight junctions, exacerbating BRB breakdown and driving the proliferation of fragile, distinctively permeable neovessels.

### 3.3 Angiopoietin dysregulation and inflammatory

Amplification Beyond the VEGF axis, the Angiopoietin-Tie (Ang/Tie) signaling pathway regulates vascular maturation and stability. Angiopoietin (Ang)-1 serves as a Tie2 receptor agonist, promoting vessel integrity. Conversely, Ang-2, upregulated in the diabetic retina, functions as a competitive antagonist destabilizing the vasculature and sensitizing endothelial cells to VEGF-induced angiogenesis (18). However, vascular destabilization is inextricably linked to a broader, chronic low-grade inflammatory cascade. Hyperglycemia-induced oxidative stress activates resident retinal immune cells—specifically microglia and Müller glia—precipitating the release of a plethora of inflammatory mediators (19).

Current evidence elucidates that this inflammatory milieu extends well beyond IL-6 and IL-1β. Tumor necrosis factor-α (TNF-α), IL-1β, and IL-6 act as primary effectors, directly compromising the blood-retinal barrier (BRB) via autocrine and paracrine signaling (20). Concurrently, the upregulation of chemokines such as CCL2 and IL-8, alongside adhesion molecules like ICAM-1, orchestrates the recruitment and adhesion of circulating leukocytes (leukostasis). This process mechanically occludes capillaries and amplifies endothelial injury via the release of proteases and reactive oxygen species (21). Furthermore, advanced glycation end products (AGEs) upregulate CD40 and potentiate CD40-CD154 signaling in retinal cells, which drives pro-inflammatory responses critical to the pathogenesis of diabetic retinopathy, a condition characterized by neurovascular degeneration (22). These mediators converge on pivotal signaling hubs, including the NF-κB and MAPK pathways, establishing a self-perpetuating inflammatory loop that drives macular edema even in the absence of active VEGF signaling (23, 24). This intricate convergence of neurodegeneration, ischemia-driven angiogenesis, and widespread inflammatory cascades underscores the limitations of VEGF-centric monotherapy.

### 3.4 Emerging pathogenic dimensions: epigenetics, microbiota, and novel signaling targets

Beyond the canonical neurovascular and inflammatory cascades, recent investigations delineate a multi-dimensional pathogenic landscape characterized by epigenetic imprinting, systemic dysbiosis, and alternative signaling pathways. Epigenetic modifications, including histone methylation and the dysregulation of long non-coding RNAs such as MALAT1 and miRNAs (e.g., targeting ROBO4), underpin the phenomenon of “metabolic memory,” perpetuating oxidative stress and transcriptional aberrations like Rac1 activation even after glycemic normalization (25). This molecular persistence is exacerbated by the “gut-retinal axis,” where intestinal barrier compromise facilitates the translocation of lipopolysaccharides (LPS) and alters the profile of metabolites like short-chain fatty acids (SCFAs) and tryptophan, thereby activating retinal Toll-like receptor 4 (TLR-4), establishing a systemic inflammatory loop (26); conversely, microbiome-derived metabolites such as tauroursodeoxycholic acid (TUDCA) act as neuroprotective agents via TGR5 receptor signaling (27). Parallel to these systemic drivers, specific ocular mediators function independently of established pathways: Secretogranin III (SCGIII) drives neovascularization through a distinct, VEGF-independent mechanism (28), while high-mobility group box-1 (HMGB1) disrupts lysosomal integrity to trigger pigment epithelial cell death (29), collectively highlighting the heterogeneity of DR progression mechanisms that evade conventional therapeutics.

## 4 Current treatment strategies of DR

The comprehensive management of DR necessitates a stratified approach that distinguishes between the systemic mitigation of risk factors, essential for retarding disease onset, and targeted ocular interventions required once pathology threatens visual integrity. Fundamental management emphasizes metabolic regulation, with evidence indicating that maintaining glycated hemoglobin (HbA1c) below 7% significantly attenuates DR incidence (30). However, in patients with established severe retinopathy, precipitous glycemic reduction necessitates caution to avert the paradoxical exacerbation of retinal progression (31). Beyond glycemic control targets, certain pharmacological agents possess neurovascular protective properties: the combination of metformin with dipeptidyl peptidase-4 (DPP-4) inhibitors is associated with reduced risk of NPDR (32). Furthermore, recent studies highlight that Sodium-Glucose Cotransporter 2 inhibitors (SGLT2i), such as luseogliflozin, may alleviate early vascular leakage and neuroglial dysfunction independent of glucose lowering (33). Regarding hemodynamic stability, maintaining systolic blood pressure ≤ 140 mmHg is associated with a lower prevalence of retinopathy (30), while the lipid-lowering agent fenofibrate has been objectively validated in major clinical trials (e.g., FIELD, ACCORD-Eye) to reduce the progression of diabetic retinopathy (34, 35). Importantly, this protective effect appears to be largely independent of lipid levels, potentially involving mechanisms such as the modulation of lipid toxicity and augmentation of circulating hematopoietic stem cells (36). Accordingly, for patients with No DR or mild-to-moderate NPDR, these systemic measures constitute the primary management strategy alongside regular ophthalmologic observation, as invasive ocular interventions are not yet indicated.

When disease progression necessitates direct ocular intervention, therapeutic strategies are stratified by clinical stage as outlined in **Table 1**. Regular monitoring remains the cornerstone for delaying visual loss, but for symptomatic diseases specifically, DME and PDR, pharmacotherapy is imperative. Anti-VEGF therapy constitutes the first-line standard of care for vision-threatening complications of DR, specifically center-involved DME and PDR-associated active neovascularization. However, limitations persist, as approximately 30-40% of patients exhibit suboptimal responses to mono-target anti-VEGF blockade (38, 39). Addressing this, dual inhibition strategies targeting both VEGF-A and Ang-2, such as faricimab, have demonstrated efficacy in extending dosing intervals and stabilizing the vasculature in DME and PDR (40, 41). For patients refractory to anti-VEGF therapy or those with pseudophakia, intravitreal corticosteroids offer an alternative mechanism by suppressing the inflammatory cascade. Options include triamcinolone acetonide, sustained-release dexamethasone implants, and fluocinolone acetonide implants; while effective in resolving edema, these agents carry a distinct risk profile, potentially inducing cataract progression and elevated intraocular pressure (IOP) (42, 43).

**Table 1:** Recommended management strategies for diabetic retinopathy (DR) by disease stage [based on the updated German clinical practice guidelines (second edition) (37)].

Disease stage	Recommended management or therapeutic approach	Evidence level/Remarks
No DR (low risk)	Systemic Management Only: Extend screening interval to 2 years (Absence of retinopathy AND systemic high-risk factors).	Focus is on risk factor control rather than ocular treatment; requires regular monitoring.
Non-proliferative DR (NPDR)	Observation: Regular ophthalmologic follow-up (Interval determined by severity).	Follow-up frequency mandates adjustment according to systemic risk profile (e.g., glycemic and blood pressure stability).
Diabetic Macular Edema (DME)		
- Without foveal involvement	Focal Laser Photocoagulation.	Recommended first-line therapy for non-center involved edema (Based on ETDRS study).
- With foveal involvement	Targeted Pharmacotherapy: Initiate intravitreal anti-VEGF agents or corticosteroids.	Requires OCT assessment; anti-VEGF necessitates multiple injections; corticosteroids indicated for pseudophakic or resistant eyes.
Proliferative DR (PDR)	Panretinal Laser Photocoagulation (PRP) as first-line therapy; Anti-VEGF as adjuvant/alternative.	PRP significantly mitigates the risk of severe vision loss; Anti-VEGF treats concurrent edema.
Severe NPDR	Consider PRP: Individualized assessment of progression risk.	Early laser intervention may be warranted in patients with poor compliance or high risk of conversion.
Intravitreal Pharmacotherapy		
- Anti-VEGF (e.g., ranibizumab, faricimab)	Indicated for DME with foveal involvement or PDR with vision-threatening complications.	Requires regular OCT monitoring; emerging dual-target agents (anti-Ang-2/VEGF) may extend durability.
- Corticosteroids (e.g., dexamethasone, fluocinolone)	Indicated for DME refractory to anti-VEGF therapy or with significant inflammatory component.	Note the risk of elevated intraocular pressure (IOP) and cataract formation.

Panretinal Photocoagulation (PRP) persists as the established therapeutic standard for high-risk PDR and severe NPDR. Mechanistically, the efficacy of PRP is attributed to the destruction of highly metabolically active photoreceptors, which downregulates the angiogenic drive by alleviating retinal hypoxia and reducing the secretion of VEGF (44, 45). While conventional continuous-wave laser provides durable suppression of neovascularization, it characterizes a destructive modality that may precipitate permanent sequelae, including constricted visual fields, nyctalopia, and reduced contrast sensitivity (46).

To ameliorate the iatrogenic sequelae associated with conventional photocoagulation, contemporary therapeutic paradigms have shifted toward tissue-sparing modalities. Foremost among these is the 577 nm subthreshold micropulse laser (SMLP), which utilizes a fractionated duty cycle to confine thermal elevation to the RPE, thereby permitting thermal relaxation and preventing neurosensory scarring (47). Validation of this non-destructive profile is substantiated by recent prospective clinical analyses comprising 24 eyes (22 patients), where standardized SMLP protocols (5% duty cycle, 50% threshold power) orchestrated significant anatomical restoration without inducing retinal damage on OCT or OCTA (47). Mechanistically, efficacy is not derived from ablation but rather through the modulation of choroidal hemodynamics; post-treatment assessments reveal a significant increase in choroidal vascular density at leakage sites, correlating with the resolution of subretinal fluid and functional recovery (47). Complementing this approach, patterned scanning systems (e.g., PASCAL) and targeted retinal photocoagulation (TRP) leverage shorter pulse durations and ultra-widefield guidance to selectively treat non-perfused peripheral retina, further maximizing the preservation of viable tissue while suppressing the angiogenic drive (44, 48).

In the era of pharmacotherapy, a critical dichotomy exists between clinical trial outcomes and real-world utility. Although large-scale trials (e.g., Protocol S) demonstrate that anti-VEGF monotherapy can achieve visual outcomes non-inferior or superior to PRP, its efficacy is contingent upon strict adherence to intensive injection protocols. In real-world scenarios compromised by economic constraints or patient non-compliance, the cessation of anti-VEGF therapy can trigger a rebound of neovascularization with catastrophic visual consequences. Consequently, PRP retains its priority as a definitive, cost-effective intervention that provides permanent prophylaxis against vision loss, particularly for patients with uncertain follow-up adherence (44, 49).

Regarding combination regimens, current evidence indicates that the routine integration of anti-VEGF and laser does not consistently yield superior visual acuity outcomes compared to pharmacologic monotherapy. Nevertheless, laser therapy maintains relevance as an adjunctive or rescue strategy to ameliorate macular edema and potentially attenuate the injection burden (50, 51), with corticosteroids serving as a viable alternative for refractory cases (52).

For advanced PDR complicated by vitreous hemorrhage, tractional retinal detachment (TRD), or combined traction-rhegmatogenous retinal detachment, Pars Plana Vitrectomy (PPV) is indicated. The surgical objectives encompass the clearance of media opacities, the relief of anteroposterior and tangential traction via the dissection of fibrovascular membranes (FVMs), and the induction of posterior vitreous detachment (53). Retrospective analyses suggest that 27-gauge vitrectomy achieves anatomical and functional outcomes commensurate with 25-gauge systems, potentially facilitating intricate maneuvers in complex detachments without necessitating bimanual techniques (54).

## 5 Anti-VEGF therapy in the management of DR

VEGF plays a pivotal role in the pathogenesis of DR. The VEGF family comprises multiple isoforms, including VEGF-A, VEGF-B, VEGF-C, VEGF-D, VEGF-E, VEGF-F, and placental growth factor (PGF). Among these, VEGF-A exhibits the most potent angiogenic activity while significantly enhancing vascular permeability, facilitating the extravasation of proteins and other molecules, thereby rendering it particularly consequential in the pathophysiology of DR and DME (4, 55). Consequently, pharmacological inhibition of VEGF binding to VEGF receptors (VEGFRs) has emerged as a first-line therapeutic strategy for DR management by targeting angiogenesis suppression to delay disease progression. Currently, anti-VEGF therapy is recommended for PDR (with or without concomitant DME), moderate-to-severe NPDR (regardless of DME comorbidity), and mild NPDR associated with DME (55). This section will provide a comprehensive overview of the clinical applications and research advancements of major anti-VEGF agents in DR treatment.

### 5.1 Bevacizumab

Bevacizumab (Avastin^®^, Genentech) is a 149 kDa humanized monoclonal IgG1 antibody specifically engineered to bind and neutralize all isoforms of VEGF-A (56). Initially approved by the FDA in 2004 for intravenous use in metastatic colorectal cancer, it was the first anti-VEGF agent to gain regulatory approval. Its application soon extended ophthalmology, with growing evidence supporting the efficacy of intravitreal bevacizumab (IVB) in managing retinal vascular diseases, including PDR, DME, and wet age-related macular degeneration (AMD), leading to widespread off-label use (5, 55).

Against this backdrop of off-label utility, the limitations of repackaged intravenous bevacizumab—including risks of microbial contamination, inconsistent dosing accuracy, and protein aggregation during aliquoting or long-term storage—have underscored the need for ophthalmic-specific formulations (57). To address these gaps, ONS-5010 (bevacizumab-vikg, Lytenava™), a dedicated intravitreal preparation of bevacizumab, has secured regulatory approval in the United Kingdom (via MHRA) and the European Union in 2024, marking the first formally authorized ophthalmic formulation of bevacizumab for retinal disorders (58). This approval was substantiated by data from the NORSE TWO trial (NCT03834753), a phase III randomized, double-masked, active-controlled study evaluating ONS-5010 in 228 treatment-naïve patients with wet AMD. In this trial, monthly intravitreal injections of 1.25 mg ONS-5010 for 12 months yielded significantly higher rates of visual acuity improvement compared to ranibizumab (administered per the PIER study regimen: 0.5 mg monthly for 3 months, then quarterly): 41.7% of ONS-5010-treated patients gained ≥15 Early Treatment Diabetic Retinopathy Study (ETDRS) letters at 11 months, versus 23.1% in the ranibizumab group (risk difference 0.1859, 95% CI = 0.0442–0.3086, p=0.0052) (59). The mean change in best-corrected visual acuity (BCVA) from baseline also favored ONS-5010 (11.2 ± 12.19 ETDRS letters vs. 5.8 ± 14.80 letters in the ranibizumab group), with complementary analyses via analysis of covariance (ANCOVA) and multiple imputation consistently confirming this superiority (p ≤ 0.0043). Importantly, the safety profile of ONS-5010 aligned with that of ranibizumab: ocular adverse events (AEs) were comparable (52.2% vs. 53.0% of patients), with conjunctival hemorrhage (8.8%) and elevated intraocular pressure (6.2%) being the most frequent ocular events in the ONS-5010 group, and only one study-related serious ocular AE (elevated IOP) reported. In the United States, however, the phase III NORSE EIGHT trial (NCT06190093) comparing ONS-5010 to ranibizumab failed to achieve its primary non-inferiority endpoint for mean BCVA change at Week 8. While subsequent 12-week data demonstrated non-inferiority (mean BCVA change +5.5 vs. +6.5 letters), the FDA issued a Complete Response Letter (CRL) in August 2025, citing inadequate efficacy evidence to support the Biologics License Application (BLA) at that juncture (60).

Beyond wet AMD, early evidence suggests potential utility of ONS-5010 in other retinal vascular diseases (e.g., DME, branch retinal vein occlusion), with its standardized manufacturing process eliminating the risks of underdosing or protein degradation associated with repackaged intravenous bevacizumab—issues that previously led to false impressions of treatment nonresponse or increased infectious endophthalmitis risk (57).

Early clinical evidence for efficacy in PDR emerged rapidly. Spaide and Fisher (61) provided a seminal report on two patients with PDR complicated by vitreous hemorrhage treated with intravitreal bevacizumab (1.25 mg) (61). Utilizing serial visual acuity testing, ophthalmoscopy, and fluorescein angiography, they documented rapid visual improvement (gains of 2 and 5 lines at 1 month), regression of retinal neovascularization, and near-complete resolution of vitreous hemorrhage within one month. While one patient required a repeat injection for persistent leakage, no adverse events were observed (61). This small study provided crucial early proof-of-concept for IVB’s efficacy in resolving PDR complications.

The safety profile of IVB has been further substantiated in larger cohorts. Wu et al. (62) conducted a large retrospective, multicenter study involving 1,173 patients (1,310 eyes) receiving IVB (1.25 mg or 2.5 mg) for various retinal disorders (including PDR and DME), totaling 4,303 injections over 12 months (mean follow-up 13.2 months). Systemic adverse events were infrequent (1.5% of patients), including hypertension (0.59%), cerebrovascular accidents (0.5%), myocardial infarctions (0.4%), and deaths (0.4%). Ocular complications were also rare: bacterial endophthalmitis (0.16% per injection), tractional retinal detachment (0.16%), and uveitis (0.09%). This extensive experience supported the short-to-medium term safety and tolerability of IVB.

A 24-month multinational retrospective analysis by Arevalo et al. specifically evaluated the long-term efficacy of IVB (1.25 mg or 2.5 mg) in 115 patients (139 eyes) with diffuse DME (63). Both dosage groups showed significant and sustained improvements in BCVA and substantial reductions in central macular thickness (CMT) over the 2-year period, with no statistically significant difference in outcomes between the two doses (63). This provided robust evidence for the sustained anatomical and functional benefits of IVB in DME management.

Theoretical frameworks postulating that the pharmacological inhibition of aqueous humor turnover—via adjuncts such as topical timolol-dorzolamide—might prolong the intravitreal half-life of bevacizumab have catalyzed investigations into combination regimens. However, recent meta-analytic evidence indicates a divergence between this pharmacokinetic rationale and clinical outcomes; specifically, aggregate data reveal no statistically significant augmentation in BCVA or CMT compared to monotherapy (64). Conversely, the clinical utility of this combination is unambiguously delineated in the management of ocular hemodynamics. While intravitreal injections are associated with transient IOP elevations, concurrent topical suppression significantly attenuates these fluctuations (64), positioning the regimen as a prophylactic strategy for IOP stabilization rather than a potentiator of retinal therapeutic efficacy.

### 5.2 Aflibercept

Aflibercept (Eylea^®^, Regeneron Pharmaceuticals) is a 115 kDa recombinant fusion protein designed as a soluble decoy receptor. It combines the key ligand-binding domains of human VEGFR1 and VEGFR2 with the Fc portion of human IgG1. This unique structure confers high-affinity binding to VEGF-A, VEGF-B, and PlGF, effectively blocking their interaction with native VEGFRs and potently inhibiting VEGF-mediated signaling pathways implicated in angiogenesis and vascular permeability (65–67). While initially approved by the FDA in November 2011 for neovascular AMD (nAMD), aflibercept’s significant impact on DR management was cemented by the landmark VISTA and VIVID Phase 3 trials. These pivotal studies provided the robust evidence leading to its FDA approval specifically for DR treatment in 2019 (67, 68). VISTA and VIVID were parallel, double-masked, randomized controlled trials directly comparing the efficacy and safety of intravitreal aflibercept (IAI) against the then-standard of care, macular laser photocoagulation, for center-involving DME (68). The studies collectively enrolled 872 patients with type 1 or 2 DM presenting with center-involving DME (VISTA: N = 466; VIVID: N = 406). Participants were randomized to receive either: 2 mg intravitreal aflibercept administered every 4 weeks (2q4); 2 mg intravitreal aflibercept administered every 8 weeks following 5 initial monthly doses (2q8); or macular laser photocoagulation. In the VISTA trial, the mean BCVA improved by 12.5 and 10.7 letters in the 2q4 and 2q8 groups, respectively, compared with only 0.2 letters in the laser group (*P* < 0.0001). Similarly, in the VIVID trial, the mean BCVA increased by 10.5 and 10.7 letters in the 2q4 and 2q8 groups, respectively, versus 1.2 letters in the laser group (*P* < 0.0001). A significantly higher proportion of eyes in the aflibercept groups achieved ≥15-letter gains compared to the laser group, and greater reductions in central retinal thickness were observed with IAI. The overall incidence of ocular and non-ocular adverse events was comparable across treatment arms. Pooled analysis of both studies demonstrated that aflibercept exhibited superior efficacy over laser therapy in both functional and anatomical outcomes. Crucially, the pooled analysis of VISTA and VIVID established that the 2q8 dosing regimen, requiring fewer injections after the initial loading Phase, was non-inferior to the more frequent 2q4 regimen in achieving these substantial functional and anatomical benefits over the study period (68).

Although the 2 mg regimen administered every 8 weeks proved non-inferior to monthly dosing, the cumulative burden of frequent intravitreal injections continues to pose logistical challenges, potentially compromising long-term adherence and clinical outcomes (69). To circumvent the limitations of frequent administration and optimize treatment durability, a high-dose aflibercept 8 mg formulation was developed to deliver a four-fold increase in molar dose, thereby prolonging the duration of effective VEGF suppression within the intraocular compartment (70). Evidence from the Phase 3 PHOTON trial (DME) and PULSAR trial (nAMD) underscores the utility of this intensified regimen. Specifically, in the DME cohort (PHOTON), aflibercept 8 mg demonstrated non-inferiority to the 2 mg dose in maintaining BCVA, while enabling treatment intervals to be extended to 12 or 16 weeks for a substantial proportion of patients without compromising efficacy (71). Anatomical assessments corroborated these findings, showing comparable reductions in central subfield thickness and retinal fluid resolution. The introduction of high-dose aflibercept signifies a potential paradigm shift towards reduced treatment burden; however, the comprehensive evaluation of its long-term safety profile and cost-effectiveness in diverse real-world populations remains a priority for ongoing clinical surveillance (69).

### 5.3 Ranibizumab

Ranibizumab (Lucentis^®^, Genentech) is a humanized monoclonal antibody fragment distinguished by the absence of the immunoglobulin Fc region associated with immune system activation, enabling specific binding to VEGF-A to inhibit its biological activity (72, 73). Owing to the structural characteristics of its Fab fragment, ranibizumab exhibits higher affinity for VEGF-A compared to the bevacizumab molecule. This structural attribute has also been postulated to confer advantages in terms of intraocular diffusion and systemic clearance (72).

Ranibizumab was initially approved by the FDA in February 2015 for the treatment of DR patients with concomitant DME, based on the outcomes of the RISE/RIDE trials (72, 74–76). Subsequently, in April 2017, this approval was expanded to encompass all forms of DR following the results of the Diabetic Retinopathy Clinical Research Network (DRCR.net) Protocol S (72, 77). A *post-hoc* analysis of the Phase III RISE and RIDE clinical trials (75) demonstrated that among 746 patients with DR complicated by DME, monthly administration of ranibizumab (0.3 mg or 0.5 mg) for 24 months, followed by a subsequent extension period allowing sham group crossover to active therapy, significantly promoted the regression of diabetic retinopathy. This effect was particularly pronounced in patients with moderate-to-severe or severe NPDR. By month 24, 78.4% and 81.1% of patients in the respective ranibizumab groups achieved a ≥2-step improvement in DR severity, versus only 11.6% in the sham group. Additionally, ranibizumab treatment reduced the probability of developing new proliferative lesions by two-thirds over the 36-month period (75). These therapeutic benefits exhibited rapid onset, sustained durability through month 36, and clinical significance, independent of baseline characteristics.

The DRCR.net Protocol S study was a randomized clinical trial designed to compare the efficacy of intravitreal ranibizumab injections versus PRP for PDR (77). The study enrolled 305 adults with PDR across 55 U.S. sites, comprising 394 study eyes. Participants were randomized to receive either intravitreal injections of 0.5 mg ranibizumab (with PRP as rescue therapy) or PRP alone, with both groups receiving ranibizumab as needed for DME. At 2 years, the ranibizumab group demonstrated a mean visual acuity improvement of +2.8 letters compared to +0.2 letters in the PRP group (difference: +2.2, 95% confidence interval: −0.5 to +5.0, noninferiority *P* < 0.001). The ranibizumab group also exhibited less peripheral visual field loss, a lower rate of vitrectomy (4% vs. 15%), and a reduced incidence of DME (9% vs. 28%). No significant differences were observed in major cardiovascular events between the two groups (77).

A *post hoc* analysis evaluated neovascular regression in the ranibizumab group over 2 years (78). At 1 month, 19% of treated eyes showed complete regression of neovascularization, with an additional 60% demonstrating improvement. By 6 months, 52% of eyes achieved regression. Over the 2-year period, 43% maintained regression, with only 3 eyes meeting criteria for treatment failure. These findings suggest that, at least within a 2-year timeframe, ranibizumab represents a viable alternative to PRP for PDR management (78).

Subsequent 5-year follow-up data revealed no statistically significant difference in visual acuity outcomes between the ranibizumab and PRP groups (3.1 ± 14.3 in the ranibizumab group vs 3.0 ± 10.5 in the PRP group) (79). However, the ranibizumab group exhibited a lower incidence of vision-impairing DME and less visual field loss. No significant differences in major systemic adverse events were observed between the two treatment arms (79).

The DRCR.net reported a randomized clinical trial comparing the efficacy and safety of intravitreous aflibercept, bevacizumab, and ranibizumab in treating center-involved DME (80). The study enrolled 660 adults with DME and randomly assigned them to receive aflibercept (2.0 mg), bevacizumab (1.25 mg), or ranibizumab (0.3 mg) injections every 4 weeks, with a primary outcome of mean change in visual acuity at 1 year. Results showed that aflibercept achieved a mean improvement of 13.3 letters in visual acuity, compared to 9.7 letters with bevacizumab and 11.2 letters with ranibizumab (*P* < 0.001 for aflibercept vs. bevacizumab and *P* = 0.03 for aflibercept vs. ranibizumab). However, the difference was driven by eyes with worse baseline visual acuity. When initial visual acuity was mild (20/32 to 20/40), there were no significant differences among the three groups. At worse initial visual acuity levels (20/50 or worse), aflibercept demonstrated a clinically meaningful advantage over bevacizumab and ranibizumab. No significant differences were observed among the groups in terms of serious adverse events, hospitalization, death, or major cardiovascular events.

Overall, the three commonly used anti-VEGF agents for retinal diseases demonstrate comparable efficacy in clinical trials. The primary determinant influencing clinical decision-making appears to be the substantial cost differential per injection (USD): according to 2022 estimates, ranibizumab at $1,883, aflibercept at $2,098, compared to the markedly lower cost of bevacizumab at merely $90 (81). In addition to the economic burden, frequent intravitreal injections impose a substantial treatment burden on patients, including pain, the risk of infection, poor compliance, and interference with daily life. Moreover, some patients do not respond well to existing anti-VEGF drugs, which highlights the necessity of identifying new targets and developing combination treatment strategies.

### 5.4 Novel therapeutic approaches utilizing anti-VEGF monoclonal antibodies

#### 5.4.1 Ranibizumab port delivery system

As previously mentioned, the substantial costs associated with frequent intravitreal ranibizumab injections may lead to treatment inadequacy among financially burdened patients. The ranibizumab-loaded port delivery system implant (PDS) (Susvimo^®^, Genentech) may potentially alleviate this treatment burden associated with intravitreal injection-based therapies. This implant comprises a drug reservoir pre-filled with RBZ that is permanently implanted in the superotemporal quadrant of the sclera. The mechanism facilitates sustained, controlled release and repeatable refilling of RBZ through a self-sealing septum (82). RBZ diffuses passively from the implant into the vitreous cavity, with the diffusion rate being concentration-dependent (82). On February 4, 2025, Roche (SIX: RO, ROG; OTCQX: RHHBY) announced that FDA granted approval for Susvimo^®^ 100 mg/mL for the treatment of DME (83).

Two Phase 3 clinical trials, PAVILION (NCT04503551) and PAGODA (NCT04108156), evaluated the safety and efficacy of Susvimo in the treatment of DR (84, 85). The Phase 3 PAVILION randomized clinical trial investigated the therapeutic efficacy of the Port Delivery System with ranibizumab, 100 mg/mL, refilled every 36 weeks (PDS Q36W) compared to conventional monitoring in patients with NPDR without center-involved DME (CI-DME) (85). One-year follow-up results demonstrated that quarterly PDS implantation achieved a higher proportion of subjects with ≥2-step DRSS improvement and reduced the risk of developing CI-DME, PDR, or ASNV compared to the control group. Regarding safety outcomes, through week 52, ocular adverse events of special interest were reported in 17 participants (16.2%) in the ranibizumab-loaded PDS Q36W arm, including severe vitreous hemorrhage and retinal detachment (85). The concurrently published Phase III Pagoda randomized clinical trial focused on DME patients, comparing the efficacy of ranibizumab-loaded PDS replenished every 24 weeks (100 mg/mL sustained delivery) versus monthly 0.5 mg intravitreal ranibizumab injections over 64 weeks (84). The results demonstrated non-inferiority of the 24-week PDS regimen compared to monthly ranibizumab injections in terms of BCVA improvement at weeks 60 and 64. However, the PDS group exhibited a higher incidence of special interest adverse events (84).

#### 5.4.2 Bevacizumab-loaded Mesenchymal stem cell-derived small extracellular vesicles (Preclinical)

The primary clinical administration route involves intravitreal injection to achieve localized drug accumulation in ocular tissues while minimizing systemic adverse effects. However, repeated intravitreal injections may lead to various ocular complications, including hemorrhage, infection, and endophthalmitis.

The development of novel drug delivery systems to reduce the frequency of intravitreal injections may mitigate ocular complications and enhance patient compliance. Mesenchymal stem cell-derived small extracellular vesicles (MSC-sEVs) have demonstrated both efficacy and safety as drug carriers in multiple clinical investigations (86). Reddy et al. engineered an innovative delivery platform utilizing bevacizumab-loaded MSC-sEVs (designated EV-BZ), reporting sustained therapeutic effects in streptozotocin-induced DR rat models (86). Experimental findings revealed that a single intravitreal administration of EV-BZ maintained reduced VEGF levels for over two months (compared to one month with free bevacizumab), while simultaneously decreasing retinal leukostasis and exudates for more than two months. Furthermore, TUNEL assays consistently demonstrated lower retinal cell apoptosis compared to conventional bevacizumab treatment (86).

The investigators concluded that MSC-derived small extracellular vesicles significantly prolong drug efficacy while preserving vitreous transparency, potentially reducing both injection frequency and associated complications in DR management.

#### 5.4.3 VEGFR-2 specifically antagonists (Preclinical investigation for DR)

Ramucirumab (Cyramza^®^, Eli Lilly) is a humanized monoclonal antibody therapeutic agent. In contrast to the aforementioned anti-VEGF monoclonal antibodies, ramucirumab specifically targeted VEGFR-2, thereby suppressing VEGF-mediated VEGFR-2 activation. This drug received FDA approval in 2014 for intravenous administration in the treatment of advanced gastric and gastroesophageal junction adenocarcinoma (87). Preclinical studies in animal models have demonstrated the safety profile of intravitreal administration at low doses (250μg) in rabbit eyes. However, higher doses (500μg) resulted in a statistically significant reduction in retinal ganglion cell counts compared to control groups when evaluated seven days post-injection (87). These findings offer preliminary validation regarding the safety profile of intravitreal ramucirumab delivery for VEGF-mediated pathologies, thereby providing a scientific basis for subsequent clinical investigations.

## 6 Therapeutic targeting of angiopoietins in DR

As previously mentioned, dysregulation of the Ang signaling pathway is associated with pathological neovascularization in the retina and disruption of the BRB, rendering Ang-2 an attractive therapeutic target for DME (88). Currently under development are monoclonal antibodies targeting Ang-2, including dual inhibitors of VEGF and Ang-2 as well as selective Ang-2 inhibitors.

### 6.1 Faricimab

Faricimab, a novel bispecific antibody, has emerged as a promising therapeutic agent in recent clinical studies (89). It consists of an anti-Ang-2 antigen-binding fragment (Fab), an anti-VEGF-A Fab, and a modified Fc region. By simultaneously targeting VEGF and Ang-2—two distinct signaling pathways implicated in retinal vascular disorders—faricimab not only effectively suppresses VEGF-mediated neovascularization but also enhances vascular stability and mitigates inflammation through Ang-2 inhibition (18, 90).

A Phase 2 randomized controlled trial BOULEVARD (NCT02699450) comparing the efficacy and safety of faricimab with ranibizumab in 229 patients with DME (91). The study included 168 anti-VEGF treatment-naïve patients randomized 1:1:1 to receive 6.0 mg faricimab, 1.5 mg faricimab, or 0.3 mg ranibizumab monthly for 20 weeks, followed by an observation period until week 36. The primary endpoint was mean change in BCVA at week 24. Results showed that 6.0 mg faricimab demonstrated statistically superior visual acuity gains over ranibizumab (13.9 vs. 10.3 ETDRS letters, *P* = 0.03), along with greater reductions in central subfield thickness, improvements in Diabetic Retinopathy Severity Scale scores, and extended durability (longer time to re-treatment during observation) (91).

Two identically designed, multicenter, randomized, double-masked, active comparator-controlled Phase 3 trials (YOSEMITE and RHINE) evaluated the efficacy, durability, and safety of intravitreal faricimab with extended dosing intervals (up to every 16 weeks) in patients (92). The two trials involved 1891 participants across 353 global sites, randomly assigned to receive faricimab every 8 weeks, faricimab via a personalized treatment interval (PTI) algorithm, or aflibercept every 8 weeks. The primary endpoint was mean change in BCVA at 1 year, averaged over weeks 48, 52, and 56. Results demonstrated non-inferiority of faricimab (both every 8 weeks and PTI) compared to aflibercept, with comparable BCVA gains (e.g., YOSEMITE: faricimab every 8 weeks 10.7 vs. aflibercept 10.9 ETDRS letters; RHINE: faricimab 11.8 vs. aflibercept 10.3 letters). Over 50% of PTI patients achieved 16-week dosing intervals by week 52, with anatomical improvements (e.g., central subfield thickness reductions) often exceeding aflibercept. Safety profiles were similar across groups, with ocular adverse events occurring in 31–43% of faricimab-treated patients (92).

Several clinical reports have documented the efficacy of faricimab in treating DME patients (93–95). A Systematic Literature Review and Network Meta-Analysis incorporated 26 randomized controlled trials (RCTs) evaluating the efficacy, durability, and safety profile of faricimab under the “treat-and-extend” (T&E) regimen (with dosing intervals extending up to 16 weeks) at the 12-month endpoint, while comparing it with other DME therapeutic approaches including anti- VEGF agents (aflibercept, ranibizumab, bevacizumab), dexamethasone, and laser therapy. The T&E protocol of faricimab demonstrated statistically superior outcomes in retinal dehydration compared to all flexible-dosing comparator agents (with 55-125 μm greater reductions in CST), numerically fewer injections (0.92-1.43 fewer administrations than other flexible regimens), while maintaining a comparable safety profile to established anti-VEGF therapies. When compared to ranibizumab/bevacizumab, faricimab exhibited significantly greater improvements in BCVA (4.4-4.8 additional letters gained), and numerically surpassed the pro re nata aflibercept regimen (by 2 additional letters) (95).

### 6.2 Nesvacumab

Nesvacumab (Regeneron Pharmaceuticals, development terminated after Phase 2 due to lack of superiority) is a fully humanized IgG1 monoclonal antibody that specifically inhibits Ang-2 (96, 97). The REGN910–3 formulation developed by Regeneron Pharmaceuticals combines a fixed dose of nesvacumab with 2 mg of aflibercept in a fixed-dose combination. This approach enables independent optimization of dosing while ensuring precise delivery of each component at its designated concentration (96–98).

A Phase 2 randomized controlled trial (RUBY study) evaluated the efficacy of intravitreal nesvacumab in combination with aflibercept versus aflibercept monotherapy in 302 patients with DME (96). The study employed a 1:2:3 randomization scheme, assigning patients to either low-dose combination (3mg nesvacumab + 2mg aflibercept), high-dose combination (6mg + 2mg), or aflibercept monotherapy (2mg) arms. The treatment regimen comprised initial dosing at baseline, week 4, and week 8, followed by various maintenance protocols through week 32. Results demonstrated that while combination therapy exhibited superior anatomical improvements (central subfield thickness reductions of -210.4μm and -223.4μm in the low- and high-dose combination groups versus -61.9μm in the monotherapy group at week 36, nominal *P* < 0.05), no significant differences in visual acuity outcomes were observed among groups (ETDRS letter gains of 6.8, 8.5, and 8.8 at week 12). Compared to monotherapy, the high-dose combination group achieved higher rates of complete central subfield fluid resolution (66.3% vs 53.7%) and improvement in DR severity scale scores (DRSS) (21.3% vs 15.2%) at week 12. Safety profiles were comparable across all groups. The concurrently conducted Phase 2 randomized controlled trial ONYX compared the efficacy of intravitreal injections of nesvacumab in combination with aflibercept versus aflibercept monotherapy in treatment-naïve nAMD patients (97). This study similarly concluded that the combination regimen failed to demonstrate superior visual acuity benefits compared to aflibercept monotherapy. These findings prompted Regeneron Pharmaceuticals to discontinue further development of nesvacumab.

### 6.3 Other multi-target agents in development

In addition to faricimab, other novel multi-targeting agents are currently under development. RO-101, a bispecific protein targeting both VEGF-A and Ang-2, has demonstrated potent binding affinity for these targets in preclinical models (39). The anti-angiogenic efficacy of RO-101 in suppressing neovascularization appears comparable to or superior to existing faricimab, while exhibiting similar or extended half-life. Animal studies have confirmed its favorable biocompatibility with retinal tissues (39).

## 7 Therapeutic applications of anti-inflammatory monoclonal antibodies in DR

The pathogenesis of DR is fundamentally underpinned by chronic inflammation, which drives the early onset of the disease and the recalcitrance of DME. Given that inflammatory responses involving endothelial damage and Müller cell activation exacerbate BRB disruption (99), targeting these pathways offers a vital therapeutic avenue.

Currently, intravitreal corticosteroids represent the clinical cornerstone for managing inflammation-dominant DR, particularly in patients exhibiting suboptimal responses to anti-VEGF therapy (100). Mechanistically, corticosteroids exert potent anti-inflammatory effects by inhibiting phospholipase A2 and the NF-κB signaling pathway, thereby broadly suppressing the synthesis of downstream mediators including prostaglandins, leukotrienes, and cytokines such as VEGF, IL-6, and ICAM-1 (101). To overcome the pharmacokinetic limitations of traditional injections, sustained-release delivery systems have been developed. The biodegradable dexamethasone implant (Ozurdex) and the non-biodegradable fluocinolone acetonide implants (Iluvien/Retisert) utilize prolonged release kinetics to maintain therapeutic vitreous concentrations for months to years, effectively stabilizing the BRB and reducing injection burden (102, 103).

However, the broad spectrum of corticosteroid activity entails significant ocular adverse events, primarily the acceleration of cataract formation and steroid-induced elevation of intraocular pressure (IOP) (104, 105). This safety trade-off necessitates the development of more precise interventions. mAbs targeting specific inflammatory cytokines or adhesion molecules have thus emerged as a promising frontier. By selectively neutralizing key pathogenic drivers while sparing homeostatic mechanisms, these agents aim to uncouple anti-inflammatory efficacy from the ocular toxicity associated with pan-suppressive steroids. The following subsections detail the preclinical and clinical advancements of these targeted biological therapies.

### 7.1 IL-6 targeted therapy

IL-6 exhibits multifaceted roles in DR pathogenesis. Research demonstrates that IL-6 not only increases vascular endothelial permeability and compromises barrier function, but under hyperglycemic conditions, DR-associated signaling pathways amplify both the intensity and duration of IL-6 activity (106, 107), Meta-analytical evidence demonstrates significantly elevated IL-6 levels in DR patients, showing positive correlation with disease severity (108), consequently emerging it as a potentially crucial therapeutic target for anti-inflammatory interventions in DR, which has garnered substantial research attention. Tocilizumab (Genentech/Roche) is a monoclonal antibody targeting IL-6, currently approved by the FDA for the treatment of rheumatoid arthritis and juvenile idiopathic arthritis (109). A series of clinical cohorts investigating noninfectious uveitis demonstrated the safety profile of systemic administration (110). The READ-4 Phase 2 study was designed to evaluate the safety, tolerability, and efficacy of ranibizumab and tocilizumab monotherapies as well as combination therapy for DME. However, due to insufficient funding, the study was withdrawn prior to initiation and no participants were ever enrolled (111).

### 7.2 IL-17A targeted therapy

IL-17A, a key cytokine secreted by Th17 cells, plays a significant role in DR pathogenesis. Beyond Th17 cells, retinal Müller cells, endothelial cells, and photoreceptors have been demonstrated to produce IL-17A. Under hyperglycemic conditions, Müller cells exhibit upregulated IL-17A expression and secretion, thereby exacerbating inflammatory responses (20, 112, 113). Clinical investigations have revealed enhanced Th17 cell activity coupled with diminished regulatory T cell (Treg) function in patients with DR, resulting in a pronounced pro-inflammatory milieu (114). This immunological imbalance may influence IL-17A expression and function through multiple mechanisms. Furthermore, elevated levels of IL-17A in both peripheral serum and aqueous humor have been clinically correlated with increased DR risk in diabetic patients (112). Animal models have demonstrated that inhibition of RORγt alleviates DR by reducing the population of pro-inflammatory Th17 cells while enhancing Treg functionality (115). These findings collectively position IL-17A as a potential therapeutic target for DR intervention. However, current clinical cohorts investigating systemic IL-17 blockade for intraocular diseases have primarily focused on noninfectious uveitis, with inconsistent outcomes regarding intraocular inflammation control across different cohorts (20).

### 7.3 TNF-α inhibitors

TNF-α represents another pivotal proinflammatory cytokine that plays a crucial role in the inflammatory response and vascular pathology of DR. TNF-α contributes to endothelial cell damage, disruption of the BRB and upregulation of VEGF expression (116, 117). Consequently, monoclonal antibodies targeting TNF-α theoretically exhibit therapeutic potential for DR.

Sfikakis et al. (118) conducted a single-center, double-blind, randomized, placebo-controlled, crossover study to evaluate the efficacy and safety of the anti-TNF monoclonal antibody infliximab in patients with DME refractory to laser photocoagulation. The study enrolled 11 patients with persistent DME and visual impairment despite two previous laser photocoagulation treatments. Results demonstrated that the infliximab-treated group exhibited significantly greater improvement in visual acuity (assessed by ETDRS letter scores) compared to the placebo group, with infliximab-treated eyes showing a 24.3% greater improvement than placebo-treated eyes (95% CI 4.8–43.7; P = 0.017). Infliximab was well tolerated. These promising findings from this small phase III study suggest that systemic or intravitreal administration of anti-TNF agents may exert therapeutic effects in DME, warranting larger-scale, long-term trials to evaluate their potential as first-line therapy for DME. Although infliximab has demonstrated modest efficacy in DME, its systemic adverse effects (e.g., increased infection risk) as a systemic antibody necessitate careful consideration.

### 7.4 IL-1β targeted therapy

In type 2 diabetes patients, IL-1β levels demonstrate positive correlations with glycemic control parameters and systemic inflammatory markers (119). Furthermore, vitreous analysis in patients with PDR demonstrates elevated IL-1β concentrations concomitant with reduced levels of IL-1Ra, the endogenous regulator of IL-1β signaling (120). Canakinumab (trade name: Ilaris^®^, Novartis Pharma Schweiz), a human monoclonal antibody targeting IL-1β, has received approval from the U.S. Food and Drug Administration for the treatment of cryopyrin-associated periodic syndromes (121). A prospective, open-label pilot study investigated the effects of systemic canakinumab (122), on retinal neovascularization in PDR. The findings revealed that subcutaneous administration of systemic canakinumab (150 mg administered three times) demonstrated no significant alteration in neovascularization associated with DR by week 24. However, promising effects were observed regarding DME, with a non-statistically significant reduction in central subfield thickness (decreasing from 313 μm to 295 μm).

Overall, based on the currently available literature, while the theoretical relevance of inflammatory targets in DR pathogenesis is widely acknowledged, clinical evidence supporting the use of anti-inflammatory monoclonal antibodies targeting these pathways remains limited in DR management. To date, no definitive clinical trial data has demonstrated significant therapeutic efficacy of targeted anti-inflammatory interventions in DR patients. This may reflect the complex role of these targets in DR pathology or indicate the need for more comprehensive investigations to elucidate their precise mechanisms and optimal therapeutic windows. Future research may require integration of sophisticated biomarker analyses to identify specific patient subgroups that could potentially respond to these anti-inflammatory therapies, thereby facilitating clinical translation of these potential treatment strategies.

## 8 Exploration of other targeted monoclonal antibodies and novel delivery strategies in diabetic retinopathy

### 8.1 Wnt pathway-targeting monoclonal antibodies

The dysregulation of the Wnt/β-catenin signaling pathway (Wnt/β-cat pathway) is considered an important factor in the occurrence and development of DR. It can induce retinal inflammation, vascular leakage, and neovascularization (123). Lee et al. (123) demonstrated that Mab2F1 (Preclinical), targeting LRP6, potently inhibits Wnt signaling (IC50 = 20 μg/mL), attenuating high glucose-induced β-catenin accumulation and overexpression of VEGF, ICAM-1, and TNF-α. *In vivo* studies confirmed its efficacy in reducing retinal vascular leakage and neovascularization in OIR rats, while also suppressing inflammatory/angiogenic factor overexpression in diabetic models. Bats et al. (124) identified Fzd7 as a regulator of vascular occlusion and neovascularization in OIR. A single intravitreal administration of either anti-Fzd7 monoclonal antibody or soluble Fzd7 receptor significantly attenuated retinal neovascularization in OIR mice, establishing the Fzd7/β-catenin signaling pathway as a therapeutic target.

### 8.2 Secretogranin III-targeting monoclonal antibodies

Distinct from pleiotropic growth factors, SCGIII has emerged as a DR-selective angiogenic ligand. Preclinical optimization of anti-Scg3 antibodies yielded EBP3 hFab (Preclinical), a candidate characterized by picomolar binding affinity (0.116 nM). Comparative efficacy studies in diabetic models demonstrate that EBP3 hFab ameliorates vascular leakage by 26.4% relative to lower-affinity variants (EBP2 hFab) and surpasses the efficacy of aflibercept by 10.3% (125). These findings underscore the potential of Scg3 inhibition to provide superior vascular stabilization with potentially fewer off-target effects on physiological angiogenesis.

### 8.3 Insulin-like growth factor-1 receptor antagonist

The crosstalk between Insulin-like Growth Factor-1 (IGF-1) and VEGF, mediated via the PI3K/Akt pathway, exacerbates pathological vascular remodeling in DR (126). While the IGF-1 receptor (IGF-1R) antagonist Teprotumumab (FDA Approved for Thyroid Eye Disease; Phase I for DME) has achieved regulatory success in other indications (127), its application in diabetic retinopathy remains undefined. Although a Phase 1 safety trial in DME was initiated (NCT02103283), the absence of published pharmacodynamic data or subsequent efficacy readouts as of 2025 highlights a stagnation in translating IGF-1R blockade to retinal therapeutics (128).

### 8.4 Bio-engineered delivery: MSC-derived extracellular vesicles

To circumvent the ocular complications associated with frequent intravitreal injections, bio-engineering strategies utilizing mesenchymal stem cell-derived small extracellular vesicles (MSC-sEVs) have been developed to extend antibody retention. The encapsulation of bevacizumab within these vesicles, designated as EV-BZ (Preclinical), creates a sustained-release platform that leverages intrinsic cellular transport mechanisms. In streptozotocin-induced DR models, a single administration of EV-BZ maintained VEGF suppression and reduced retinal leukostasis for over two months—doubling the duration of efficacy compared to free bevacizumab (86). Furthermore, the significant reduction in retinal cell apoptosis observed with this platform highlights the dual benefit of prolonged therapeutic exposure and enhanced biocompatibility.

## 9 Current challenges in monoclonal antibody therapy for diabetic retinopathy

Although intravitreal injection of monoclonal antibodies, particularly anti-VEGF agents, has been established as an effective approach for DME and DR, certain limitations persist in their clinical application, especially regarding DR treatment. The primary concern relates to cost-effectiveness considerations.

A 2024 discrete-event simulation-based cost-effectiveness analysis compared anti-VEGF agents with PRP for treating PDR in the UK, modeling their lifetime impacts on patients’ visual acuity, DME, and vitreous hemorrhage (129). The findings demonstrate that anti-VEGF therapy is unlikely to be cost-effective compared to PRP, typically incurring higher costs while yielding comparable health outcomes. For every additional £3,688 spent on anti-VEGF treatment, there was a reduction of 0.029 quality-adjusted life years (QALYs), resulting in a net health benefit of -0.214 at a willingness-to-pay threshold of £20,000. Scenario analyses suggest that anti-VEGF agents may only demonstrate cost-effectiveness potential under highly specific conditions, with remaining uncertainties regarding their long-term efficacy and treatment adherence. Regarding the potential benefits of combined anti-VEGF and PRP therapy, a meta-analysis incorporating 23 randomized controlled trials (up to June 2022) demonstrated that while the combination therapy may yield superior visual acuity outcomes compared to PRP monotherapy, the magnitude of improvement did not reach clinical significance (130).

Moreover, clinicians must be cognizant that repeated intravitreal injections and their substantial financial burden contribute to poor treatment adherence among patients. A study reported that during an approximately 4-year follow-up period after PRP or intravitreal anti-VEGF injections, a considerable proportion of PDR patients were lost to follow-up (LTFU), with 22.1% of those receiving anti-VEGF therapy and 28.0% of PRP-treated patients becoming LTFU (131). Although the LTFU rate was relatively lower in the anti-VEGF group compared to PRP, subsequent investigations revealed that PDR eyes receiving intravitreal anti-VEGF therapy with LTFU exceeding 6 months demonstrated significantly worse anatomical and functional outcomes than their PRP-treated counterparts (132).

Furthermore, not all DR patients benefit from anti-VEGF therapy. A subset of patients demonstrates suboptimal response to existing anti-VEGF agents, commonly referred to as “non-responders,” or develops treatment resistance over prolonged therapy, resulting in diminished therapeutic efficacy (133). This phenomenon may be attributed to the intricate pathophysiology of DR, wherein VEGF represents only one of multiple pathogenic factors, while sustained activation of alternative pathways (e.g., Ang/Tie signaling and inflammatory cascades) may contribute to disease progression.

## 10 Conclusion

DR remains a major global cause of visual impairment, and this review summarizes the progress of mAb-based therapies. Anti-VEGF monoclonal antibodies constitute the cornerstone of pharmacotherapy for DME and PDR, delivering robust functional recovery with a manageable safety profile. Comparative analyses indicate that aflibercept demonstrates superior visual gains specifically in eyes with worse baseline visual acuity, whereas bevacizumab offers a distinct cost-utility advantage for mild cases over the costlier licensed agents ranibizumab and aflibercept. New delivery systems like ranibizumab’s port delivery system offering sustained release, while preclinical bio-engineered solutions like bevacizumab-loaded extracellular vesicles show potential to reduce injection frequency and improve adherence. Bispecific antibodies such as faricimab, targeting VEGF and Ang-2, achieve good results with longer dosing intervals, but nesvacizumab’s failure shows the need to optimize multi-target strategies. Inflammatory mediators like IL-6 and IL-1β are potential targets, but their mAb-based therapies need more trials.

Despite these advancements, critical challenges persist. Cost-effectiveness analyses indicate that anti-VEGF therapies may not outperform PRP in long-term health outcomes, particularly in resource-limited settings. Additionally, repeated injections and poor treatment adherence remain barriers, with lost-to-follow-up rates associated with worse anatomical and functional outcomes. Long-term safety, including systemic cardiovascular risks and ocular complications (e.g., endophthalmitis), demands further investigation. Future research should focus on three key areas: (1) developing long-acting drug delivery systems to minimize injection frequency; (2) refining multi-target strategies to synergize anti-angiogenic, anti-inflammatory, and neuroprotective effects; (3) establishing personalized treatment algorithms based on disease stage, baseline characteristics, and molecular biomarkers to optimize efficacy and reduce costs. Moreover, exploring novel targets beyond VEGF and Ang-2, such as oxidative stress and mitochondrial dysfunction, may expand therapeutic options for refractory cases. In conclusion, monoclonal antibody-based targeted therapies have revolutionized DR management, offering precise and effective alternatives to conventional interventions. Addressing current limitations through innovation in drug design, delivery, and personalized medicine will be pivotal in reducing the global burden of DR-related blindness.
